# 

**Published:** 1882-03

**Authors:** 


### MICHIGAN DENTAL ASSOCIATION.

The twenty-seventh annual meeting of the Michigan State Dental Association will be held in Detroit, at the Russell House, beginning March 29 at 7½ o'clock P. M., and continue ten days.

At the last meeting of the Society it was decided to make the coming meeting one of unusual interest, by introducing exercises appropriate to the celebration of the quarter-centennial of its existence. One feature of special interest will be the presentation of a history of dentistry in Michigan. Another will be the exhibition and reunion of all the charter members now living, so far as possible. This, with the stories and experiences of dentistry in the early days of Michigan, will render the meeting one of unusual interest. It is expected that all the representative men of the profession in the State will be present, and many from other States have given assurance of their intention to be present.

And that the occasion may be one of the fullest enjoyment, a superb banquet will be given on Friday evening, under the supervision of the proprietor of the Russell House. Nothing more need be said here, as to what that will be; but come and see.

The following programme of subjects for papers and discussions, is presented, viz.:

1. Treatment of Deciduous Teeth.
2. Treatment of the first permanent Molar.
3. Methods of Mounting Artificial Crowns on Natural Roots.
4. Transplanting and Replanting of Teeth.
5. Oral Surgery.

The work upon these subjects, together with other matters that will occupy the attention of the meeting, will make it necessary to economize the time very closely.

This will doubtless, in many respects, be the most interesting meeting of this body ever held, and every dentist in Michigan, who has any pride or interest in his profession, should be present. Then let all come.

### THE DENTAL REGISTER,

A MONTHLY MAGAZINE DEVOTED TO THE INTERESTS OF THE

**DENTAL PROFESSION.**

*Terms Reduced to $2.00 Per Year. Single Copies, 25 Cents.*

**EDITED BY PROF. J. TAFT, D.D.S.**

AND

***Published by SPENCER & CROCKER, Ohio Dental Depot.***

The volume commences in January and closes in December.

The REGISTER being issued monthly is always fresh, therefore Indispensable to the practitioner who desires to keep posted up with the new inventions and improvements which are daily developed. Neither expense nor effort will be spared to give value and variety to its contents Articles will be illustrated with well executed engravings whenever they will add to the interest or assist to explanation.

Its extensive circulation and frequency of issue make it one of the BEST DENTAL ADVERTISING MEDIUMS in the United States.

All subscriptions must begin with January or July.

Local or special notices, five lines or less, will be inserted for $3 each insertion; over five lines, 50 cts. per line.

All advertising bills payable in advance, or at furthest, after the issue of the first number containing the advertisement. All transient advertisements to be paid for in advance.

☞CONTRIBUTIONS SOLICITED.

Exclusively Editorial Communications should be addressed to the Editor.

The latest number of the REGISTER may be ordered with or without other goods at any time, and all letters should be addressed to

**SPENCER & CROCKER, Ohio Dental Depot, Cincinnati, Ohio.**

